# *Ailanthus altissima* Forests Determine a Shift in Herbaceous Layer Richness: A Paired Comparison with Hardwood Native Forests in Sub-Mediterranean Europe

**DOI:** 10.3390/plants9101404

**Published:** 2020-10-21

**Authors:** Silvia Montecchiari, Giulio Tesei, Marina Allegrezza

**Affiliations:** Department of Agricultural, Food and Environmental Sciences, Marche Polytechnic University, I-60131 Ancona, Italy; g.tesei@pm.univpm.it (G.T.); m.allegrezza@staff.univpm.it (M.A.)

**Keywords:** *Ailanthus altissima*, invasive alien species, photosynthetic active radiation, nemoral species, soil sampling, paired comparison, community

## Abstract

*Ailanthus altissima* is an invasive alien species (IAS) present throughout Europe and included in the list of alien species of Union concern. In sub-Mediterranean areas of central Italy, there is a lack of knowledge about this invasive species and its interactions with the native forest ecosystems. We aim to find what are the main differences in vegetation structure and floristic diversity between *A. altissima* forests and native forests through the assessment of the principal ecological parameters that differ between the forest types. We performed 38 phytosociological relevés and sampling of ecological parameters in *A. altissima* forest communities and neighboring native forests. We analyzed how species richness, diversity, life forms, life strategies, structural characteristics, and ecological parameters changed in *A. altissima* forests compared with native ones. We found that in *A. altissima* forests, there is a shift in herbaceous layer richness, with a higher presence of annual ruderal herbs and the absence of herbaceous species linked to the forest environment. The ecological parameters that diverge from the native forests were total nitrogen, total carbon, and C/N ratio. *A. altissima* forest communities could threaten the biodiversity of the native forest ecosystems in the sub-Mediterranean landscape, favoring ruderal species and inhibiting the presence of typical forest species.

## 1. Introduction

The worldwide spreading of plant species outside their native range [1] is becoming an expansive phenomenon [2,3]. Invasive alien species (IAS) are one of the greatest threats to global biodiversity and the sustainable functioning of ecosystems [4] and, for this reason, are subject to a strict policy regulation such as the EU Regulation 1143/2014 on invasive alien species [5]. *Ailanthus altissima* is one of the most important IAS in Europe, also due to its wide presence [6]. In the recent commission implementing regulation (EU), 2019/1262 of 25 July 2019 [7] the list of IAS of Union concern was updated and *A. altissima* was inserted following a process of risk assessment. This species was first introduced in Europe from China, in the 1740s, in Paris, used as ornamental plants in cities and for afforestation [8]. Nowadays, it shows a wide diffusion in urban and peri-urban areas, but also in the agro-forest environment [8,9,10,11]. In Italy, it appeared in 1760 when it was introduced at the Botanical Garden of Padua [12], and now it is considered as invasive in all Italian administrative regions [13,14], exerting an ecological impact on plant communities and Natura 2000 habitats [15]. The expansions of *A. altissima* is due to its pioneer characteristics such as the efficacy in gamic reproduction and dissemination [16,17], agamic reproduction with strong sprouting ability also through radical activity [18,19,20], and extremely rapid growth [19] that can easily outcompete the forest native species, e.g., [21,22,23]. There is a species-specific mechanism in the invasion of recipient habitats. *A. altissima* colonization probabilities are higher in hardwood forests that are dominated by the trees of the genera *Fraxinus*, *Quercus,* and *Ulmus* in the floodplains and hill positions and in dry conditions [22]. Moreover, the production of allelopathic substances (e.g., ailanthone) from the cortex and leaves that accumulate in the soil can inhibit the germination of native species [24,25]. *A. altissima* spontaneous secondary stands also have impacts on soil properties and nutrient cycling, which are fundamental components of ecosystem functioning and processes. *A. altissima* can modify carbon and nitrogen cycling [26,27,28] thanks to the decomposition rates of the leaf litter [29], and alter the soil pH, even though the mechanisms are elusive [30]. Although its wide distribution all over the Mediterranean and temperate Europe, where it is mainly confined to cities at northern outposts of its range [19], there is a lack of floristic-vegetational studies along with ecological studies in a paired comparison with the reference forest in its meridional range in sub-Mediterranean and Mediterranean bioclimates.

The aims of this study were to compare floristic-vegetational and measured ecological data in mature *A. altissima* forest coenoses and neighbor native forests as reference. Thanks to this comparison, we want to highlight the effects of the alien forests canopy on the herbaceous layer, in terms of diversity and ecological conditions with respect to the native forests.

Specifically, we want to (i) identify and investigate floristic-vegetational and ecological parameters of *A. altissima* forest communities in sub-Mediterranean areas of central Italy (ii) investigate light, temperature, and soil parameters that mainly characterize those communities; (iii) investigate differences in species diversity and composition of vegetation layers and differences in habitat conditions through a paired comparison with native forests.

## 2. Results

### 2.1. Community Floristic Diversity

**Figure 1:**
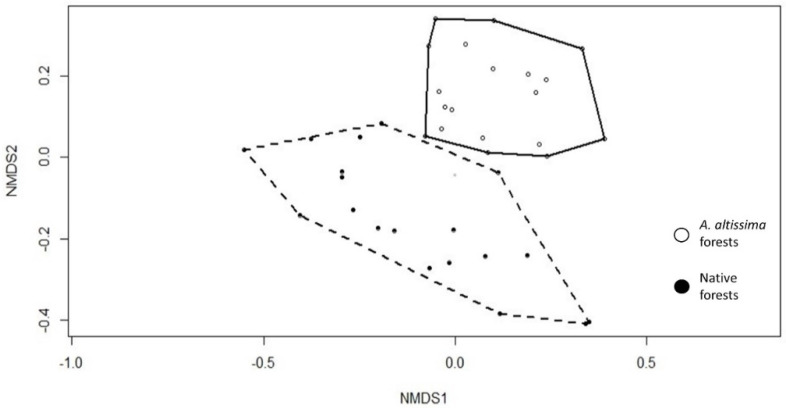
Nonmetric multidimensional scaling (NMDS) ordination diagram (axes NMDS1 and NMDS2) of the *A. altissima* and reference forest plots. The stress value of the ordination is 0.15.

From the Nonmetric multidimensional scaling (NMDS) ordination plot (Figure 1), two principal directions of variation, in term of floristic composition, are distinguished that allows us to identify two groups, albeit with very different internal variability (NMDS stress 0.15).

**Figure 2:**
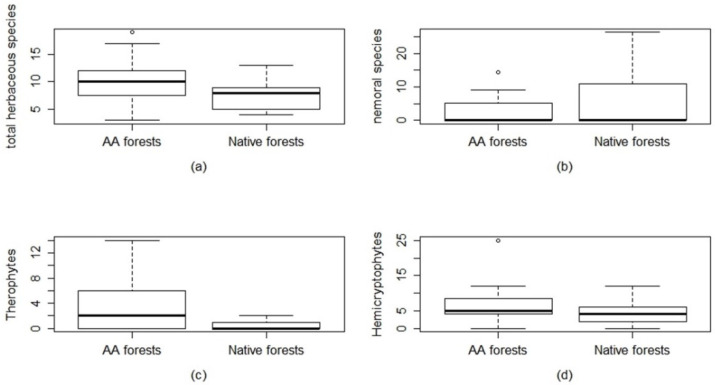
Comparison of the herbaceous layer diversity and life forms between *A. altissima* (AA forests) and Native forests. (**a**) total herbaceous species diversity (*p* value = 0.05 *), (**b**) nemoral species diversity (*p* value = 0.065), (**c**) therophyte species (*p* value = 0.006 **), (**d**) hemicryptophyte species (*p* value = 0.085). *p* level: *** *p* < 0.001; ** *p* < 0.01; * *p* ≤ 0.05. Significant values are in bold.

There are two groups, corresponding to the two forest types under study: plots dominated by *A. altissima* (group 1) and plots dominated by *Quercus pubescens*, *Populus nigra*, or *Ulmus minor*, representing the native forests (group 2). Indicator species analysis (IndVal) revealed the species associated with each of the two groups. The indicator species of group 1 are *A. altissima* (Mill.), *Chaerophyllum temulum* L., *Sambucus nigra* L., and those of group 2 are *Q. pubescens* Willd, *Ostrya carpinifolia* Scop, *U. minor* Miller. So, the groups correspond to the different forest coenoses types under study: native forests dominated by the typical forest’s species of the study area and the alien *A. altissima* forest communities. Shannon diversity indices of the vegetation layers were compared between the two forest types and resulted in not being significant. Species diversity in terms of the number of species (richness) shows a significant difference only for the herbaceous layer (Table 1). As shown in Figure 2 *A. altissima* forests have higher species richness in the herbaceous layer than native forests. 

### 2.2. Herbaceous Layer: Species Diversity, Life Forms, and Grime Strategies

The species diversity analysis performed for each vegetation layer suggests that the main differences are at the level of the herbaceous layer. The life form distribution (Figure 2) shows that there are more geophytes in the native forests (data not shown) such as *Teucrium chamaedrys* L., *Cyclamen hederifolium* Aiton, *Cyclamen repandum* Sm., *Carex flacca* Schreber, *Helleborus bocconei* Ten. *A. altissima* forests communities show a greater presence of hemicryptophytes and therophytes (annual herbaceous species) with respective *p*-values of 0.08 and <0.01 (Figure 2).

**Figure 3:**
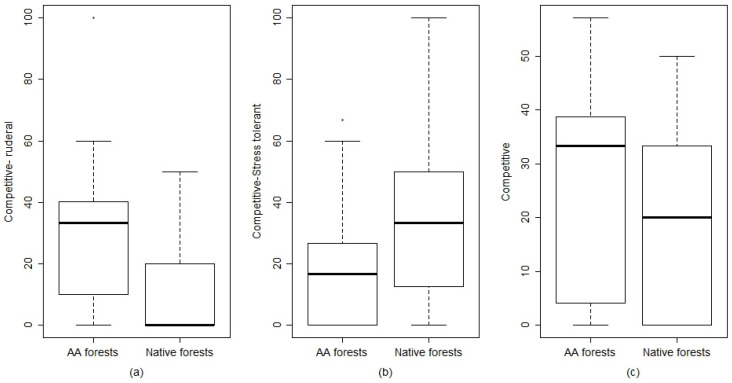
Comparison of the significant Grime strategies between *A. altissima* (AA forests) and native forests. (**a**) Competitive-ruderal *p* value = 0.008 **; (**b**) Competitive-stress tolerant *p* value = 0.005 **; (**c**) Competitive *p* = 0.1. *p* level: *** *p* < 0.001; ** *p* < 0.01; * *p* ≤ 0.05. Significant values are in bold.

Specifically, in *A. altissima* herbaceous layer, the hemicryptophytes species represent almost 43% of the *A. altissima* forests herbaceous layer. Those species are biennial or perennial, frequently linked to open environment or nemoral/forest edge species, linked to forest environments (Table S1). Therophytes are heliophilous annual species that represent almost 35% of the *A. altissima* forests’ herbaceous layer (Table S1). From the total herbaceous species, we analyzed the nemoral component (Figure 2). In terms of weighted percentage cover, the nemoral component is linked to the native forest and is statistically significant (*p*-value = 0.1) (Figure 2 and Table S1). Regarding the Grime life strategies (Figure 3) in the herbaceous layer, the *A. altissima* community shows a greater presence of species with a competitive-ruderal strategy (CR, *p* = 0.008) that are species adapted to disturbances as well as species having a competitive strategy (C, *p* = 0.1). In the native forests, it was found a greater presence of species having a competitive-stress tolerant strategy (CS, *p* = 0.005).

### 2.3. Ecological Variables

**Figure 4:**
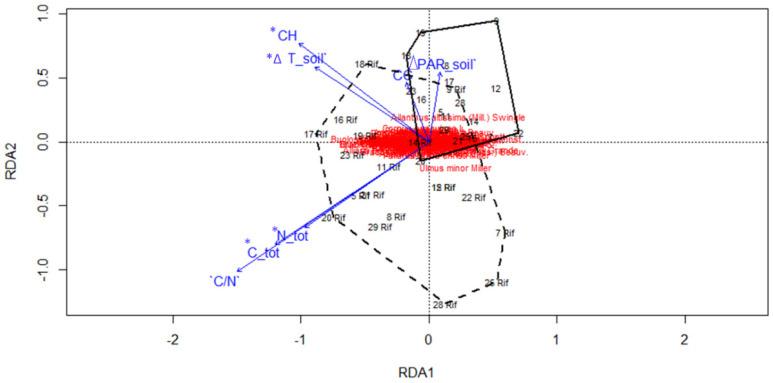
RDA Ordination diagram of vegetation plots and species in relation to the considered environmental variables. *A. altissima* forests are within the dashed line. Native forests are within the continuous line. Significant variables (at *p* value ≤ 0.05) are reported with asterisks. RDA1 explains 39.1% of the cumulative amount of variance expressed as proportions of the total explained variance and RDA2 axes as 19.02%. For abbreviations of the variables, see Table 3.

In order to identify the trends of environmental variables in relation to the two forest vegetation types, an RDA was performed (Figure 4).

**Figure 5:**
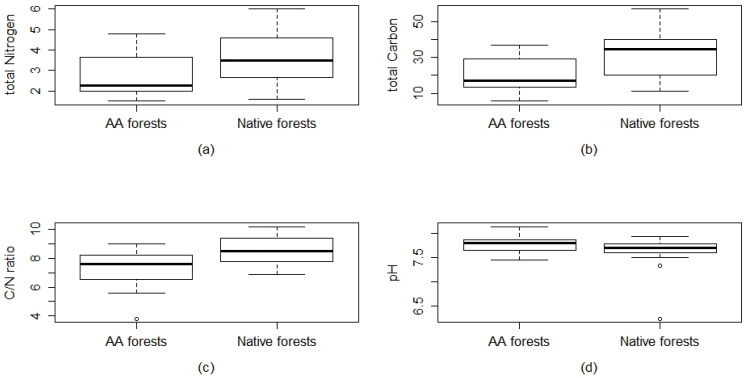
Comparison of the soil chemical parameters measured for the *A. altissima* and native forests through box plots. (**a**) Total nitrogen, *p* value = 0.025 *; (**b**) Total carbon, *p* value = 0.007 **; (**c**) carbon/nitrogen ratio, *p* value= 0.003 **; (**d**) reaction expressed in pH in H2O, *p* value = 0.1. *p* level: *** *p* < 0.001; ** *p* < 0.01; * *p* ≤ 0.05. Significant values are in bold.

The results of the RDA reveal, among the set of environmental variables, that canopy height, ΔT soil, C tot, and N tot are significant variables (Table 2). This proves that those factors are the main environmental variables shaping the plant community. The total model shows a significance level of *p* = 0.001, the constrained variation represents 24% of the total variation. The cumulative amount of variance expressed as proportions of the total explained variance (24%) by RDA1 axes is 39.7% and RDA2 axes 19.02% (total of 59.02%) To better observe the variation of environmental variables in relation to the two groups and test their statistical significance, box plots were generated. The difference between the averages of measured environmental values such as Canopy height, canopy cover, ΔPAR, and ΔT were all non-significant in the comparison between the two forest types. A slight trend between a narrow range of values can be observed in the ΔPAR as well as the canopy-cover, that shows higher average values in the *A. altissima* forests (Table S2). In the first case, this means the presence of a greater difference between the radiation recorded outside (full light) and the one recorded below the forest canopy. The comparison of the measured edaphic characteristics is shown in Figure 5. The pH values were not different between the groups. Total nitrogen, total carbon and C/N ratio were significantly different between the two groups (pH *p* = 0.1; N tot *p* = 0.025 *; C tot *p* = 0.007 **; C/N *p* = 0.0033 **) with *A. altissima* forests having lower values for all three variables considered.

## 3. Discussion

### 3.1. Species Diversity, Life Forms, Grime Strategies

The results show that *A. altissima* forests host a pool of species that diverge from the target species of the compared native forests. In opposition to a recent study on other alien forest species [31], we found a lack of species linked to nutrient-rich conditions. The species richness of the tree and shrub layers seems to not determine a significant differentiation of the two coenoses. In accordance with several other studies in invasion ecology, the main effect is found in the herbaceous layer, e.g., [30,32]. Floristic diversity (Richness) was higher in *A. altissima* forests in comparison to the native forest coenoses. Even if Constan Nava et al. [33], Motard et al. [17], Vilà et al. [26] reported a reduced species richness in plant communities dominated by this alien species, our result is consistent with the findings of Fotidias et al. [21]. The latter research reports a reduced species diversity in the herb layer in *A. altissima* coenoses in comparison with *Q. pubescens* dominated forest vegetation in a Mediterranean environment. Our result indicates that this alien species invades different environments with a human-mediated process, from seminatural and ruderal habitats to abandoned arable land. Its wide tolerance in different habitat types favored the presence of perennial and annual grass species (hemicryptophytes and therophytes) in the invaded forests. Ruderal species are those species that thrive on high disturbance levels and do not tolerate a high level of stress [34]. This ecological strategy group comprehends mainly hemicryptophytes (perennial) and therophytes (annual) life forms. The significant higher presence of annual and perennial herbaceous species, along with ruderal species in *A. altissima* plots, can be explained by the presence of altered vegetative conditions. Both of those elements are reliable indicators of disturbed conditions [35]. In *A. altissima* forests coenoses, there is the total absence of the herbaceous nemoral species characteristics of the native forest ecosystems of the study area. We found that *A. altissima* could change the species composition of the herb layer, through direct competition for resources and modification in local environmental conditions. The presence of the herbaceous nemoral component is known to be an important element for habitat identification and quality assessment compared to invaded ecosystems, e.g., [36]. Its total absence, even if in suitable environmental and landscape conditions, could be due to the presence of phytotoxic substances (ailanthone) contained in *A. altissima* leaves and bark, which can accumulate in the soil [25]. It was demonstrated that the spontaneous herbaceous species, more specifically nemoral species that are frequent under the native forest canopy, are susceptible to the presence of allelopathic compounds [17]. The presence of these phytotoxic substances leads to a reduction of germination success of the spontaneous nemoral species [24] and if this toxic compound accumulates for a long period, it could have a strong impact on the resident plant community.

### 3.2. Ecological Variables

The strongest difference between the two forests communities was found for the topsoil parameters. Specifically, the results of the edaphic characteristics consistently indicated the capability of this invasive species to alter some soil properties. Our results are inconsistent with other studies relating to the effects of *A. altissima* on the soil in the Mediterranean environment [26,27]. According to Medina-Villar et al. [37] and Castro-Díez et al. [28,38], we found lower total nitrogen and carbon and C/N ratio in *A. altissima* plots. Castro-Dìez et al. [28] observed that the lower presence of total nitrogen and C/N ratio in *A. altissima* forests is in correspondence to the maximum nitrogen mineralization activity in the same period of the year in which our survey was carried out. It was demonstrated that in invaded ecosystems, the net N mineralization and nitrification rates were almost 50% higher than in the native ones. This modification is due to the quality of the alien species litter that can alter the decomposition rates, accelerating the ecosystem nutrient cycling processes [39]. In accordance with Lazzaro et al. [40], a lower C/N ratio in invaded ecosystems could be linked to the quality of organic matter under *A. altissima* canopy and a consequent shift in soil bacterial community. A high C/N ratio means high litter quality with topsoil rich in organic matter. The main driver in C/N ratio levels is tree species, and Mediterranean oak forests have higher C/N values with respect to other invasive species forests [41]. *A. altissima* forests showed no significant impact on soil pH, in accordance with Vilà et al. [26] and Castro-Díez et al. [28].

Regarding the other environmental variables, we found that canopy height significantly contributes to explaining part of the species compositions of the two forests coenoses, in fact, this variable is linked to forest permanence. Moreover, we can assume that there are altered light conditions under *A. altissima* forests canopy. We found higher Δ PAR under *A. altissima* forest canopy than in native forests, mainly due to the presence of a dense layer of *A. altissima* renovation. *A. altissima* is known to be a species adapted to full light conditions [19], but on the contrary to what is assumed for invasive alien species, it has been demonstrated that even in low light conditions, *A. altissima* is able to reproduce with good survival rates [42]. These factors lead to the alteration of the herbaceous layer species composition and diversity under the alien species canopy. In the present study, we highlighted the principal floristic and environmental differences between *A. altissima* forests coenoses and native forests typical of the hilly landscape of sub-Mediterranean bioclimate in southern Europe. This work is the first contribution to a specific characterization of *A. altissima* forest coenoses in Italy and, from a broader point of view, for southern Europe. In the light of the recent introduction of *A. altissima* in the list of invasive alien species of European Union concern [7], a better understanding of the ecological behavior of this species is essential. 

## 4. Materials and Methods

### 4.1. Study Area

**Figure 6:**
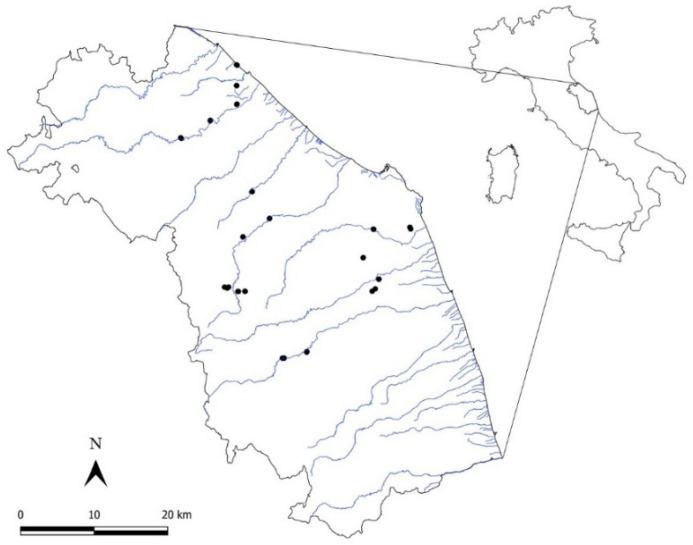
Location of the study area in sub-Mediterranean central Italy, showing the distribution of the paired sampling units. Each pair of sampling is represented by a single black point.

The forest coenoses were investigated in the sub-Mediterranean region of central Italy (Figure 6) at altitudes between 10 m a.s.l. and 500 m a.s.l., and the prevailing lithotypes are pelitic–arenaceous, arenaceous–pelitic, and alluvial. The study area is characterized by a macrobioclimate that ranges from Mediterranean, pluviseasonal oceanic bioclimate, and upper mesoMediterranean thermotype to the Temperate sub-Mediterranean variant, oceanic bioclimate, and lower meso-temperate thermotype [43], according to the bioclimatic classification sensu Rivas-Martınez [44]. According to the level 3 CORINE Land Cover 2018 (CLC) [45], the principal land-cover/land-use types of forest coenoses, are non-irrigated arable land (code 211) and heterogeneous agricultural areas with complex cultivation patterns (code 242), along with areas occupied by agriculture, significant areas of natural vegetation (code 243) and artificial surfaces (codes 112, 113, 141). The plant landscape mainly consists of crops, agro-forest environments, and native forest vegetation, such as oak forests on slopes (*Q. pubescens, Q. virgiliana*) (alliance *Carpinion orientalis*, class *Querco roboris-Fagetea sylvaticae*), riparian woods of *Salix alba* and *P. nigra* (alliance *Populion albae*, class *Salici purpurae-Populetea nigrae*) and *U. minor* communities (alliance *Lauro nobilis-Ulmion minoris*, class *Salici purpurae-Populetea nigrae*) [46].

### 4.2. Sampling Design

One method of investigating the effects of alien compared to native stands is to use nearby paired sampling units, invaded and non-invaded. As proven by Bazalova et al. [47], “the twin plots method proved to be a suitable tool for analyzing the impact of alien trees on understory vegetation”. The target forest communities were identified following complementary techniques: the consultation of the Italian National Forest Inventory [48] data on forests type distribution (following the distribution of the category “Robinio-Ailanteti”), photo-interpretation of 2012 geo-referenced images available on the “Geo-portale Nazionale” (http://www.pcn.minambiente.it), expert assessment, Google Street View that provided additional data on the presence of *A. altissima* forests growing along roads [49], and reconnaissance days in the study area (in early spring of 2019). The identification was first applied to *A. altissima* forests communities, then, using the same method, we detected native forest communities setting a circular buffer area within a 500 m radius from the detected alien forest community [50]. The native forests communities are *Q. pubescens*, *P. nigra,* and *U. minor* dominated forests and represent the typical spontaneous forest vegetation for the study area. This method assures homogeneity in terms of land-use context and disturbance regime for both alien and native stands. This is important to make comparisons that allow us to understand if and how the presence of *A. altissima* influences the ecological variables under the canopy and the vegetation composition and diversity.

### 4.3. Vegetation Survey

We applied a pairwise sampling technique to conduct a comparative study in the southern European limit of this IAS. We identify 19 couples of *A. altissima* dominated forests and native forests. A total of 38 phytosociological relevés (vegetation plots) were performed in a non-tree-lined row to avoid edge effects and detect only the real influence of the alien species on the understory layer, within an area of at least 100 m^2^, with a clear dominance of *A. altissima* or the native forests species (Braun-Blanquet cover-abundance values from 3 to 5), with an average age of at least 20 years assessed by photo-interpretation of 1994, 2000, 2006 geo-referenced images available on the Geo-portale Nazionale (http://www.pcn.minambiente.it/GN/accesso-ai-servizi/servizi-di-visualizzazione-wms). The vegetation survey was conducted according to the phytosociological methods [51]. In each plot, for each vascular plant taxa recorded according to the vegetation structure, a cover-abundance value was given, following a seven-grade scale of abundance and dominance [52]. The vegetation layers considered were three, defined as tree layer (height > 7 m), shrub layer (height 20 cm–7 m), and herb layer (height < 20 cm). The nomenclature of the vascular species follows the check-list of Italian flora [53] and the check-list of the vascular flora alien to Italy [13]. The life forms of the species follow Flora d’Italia [54]. Data on Competitive, Stress-Tolerant and Ruderal (CSR) strategies sensu Grime [33] for the herbaceous species were partly available from the online databases BiolFlor [55], database of the Czech flora and vegetation [56], and from Hunt et al. [57]. For 8 taxa, information on the CSR strategies follows specific literature reference: *Bellevalia romana* (L.) Sweet from Astuti et al. [58], *Arundo donax* L., *Asparagus acutifolius* L., *Rubia peregrina* L., *Olea europaea* L., *Orobanche minor* Sm., *Pistacia terebinthus* L. from Benhamiche-Hanifi et al. [59], *Umbilicus horizontalis* (Guss.) DC. from Bocchieri and Iiriti [60]. For the identification of the nemoral species of each relevé, we considered the syntaxonomic attribution of the species, according to the Prodrome of the Italian Vegetation [61], as present on the updated site of the Italian Botanical Society (http://www.prodromovegetazioneitalia.org/), with references to that of the European vegetation [62]. Nemoral species are those herbaceous species that are diagnostic of forest classes according to the syntaxonomic system, e.g.,: *Querco roboris-Fagetea sylvaticae* class. Those herbaceous species, expressed in weighted percentage values for each plot, are linked to forest environments and are indicators of environmental quality.

### 4.4. Sampling of the Ecological Variables

We performed field measurement of a set of ecological variables (Table 3) in both *A. altissima* forests and native forests. 

According to invasion ecology literature [11,26,32], we selected those variables that show high explanatory capability. Canopy cover (percentage of the sky covered by leaves) was measured by the mobile application GLAMA [63]. Data on canopy cover were measured in the field for all 19 twin vegetation plots. In each plot, one measurement was taken at the center of the plot of 100 m^2^ as a representative, at 1.30 m from the soil surface with a hemispherical lens. Photosynthetically active radiation (PAR) was measured in each plot between September and October of 2019 with a photo-radiometer (Delta OHM, HD 2302.0, Milan, Italy). We took four randomly PAR measures per plot, in the Native and *A. altissima* plots, respectively, from 10:00 a.m. to 5:00 p.m. at 1.30 m (PAR30) and 20 cm above the soil surface (PARsoil). We also performed four measurements outside the forest canopy (PARout) in conditions of full light to calculate the ΔPAR (difference from outside full-light conditions and inside forest canopy conditions ΔPAR30 = PARout-PAR30; ΔPARsoil = PARout-PARsoil). The difference between the inside forest and outside were made to minimize the effects of the different light conditions due to the sun height in the sky at different hours on a day. The height of the forest canopy was taken by means of 1 measurement of the dominant tree using an optical height meter (PM-5/360 PC model; Suunto Instrument Co., Helsinki, Finland). The soil was randomly collected from three different subplots and subsequently pooled for each plot. For each sample, surface litter (if present) was removed and the top 20 cm of soil was sampled (from 0 to 20 cm depth). Samples were analyzed for nitrogen (N tot), total extractable carbon (C tot), and pH in H_2_O. The ratio of total extractable carbon to total nitrogen (C/N) was also calculated as an estimate of soil quality. Soil samples were analyzed by the Marches Region agrochemical analysis and research laboratories according to the methodological standards established by Italian ministerial decree 13/09/99. Total nitrogen was extracted through Kjeldahl mineralization with hydrogen peroxide and determination of total nitrogen by distillation according to Kjeldahl; for the total extractable carbon is followed the extraction, fractionation (by means of solid-phase adsorption chromatography) and determination of organic carbon procedure reported in the ministerial decree; pH in H_2_0 was determined by potentiometric method, after calibration of the measurement system, on suspensions of soil-water.” The topsoil temperature was detected using a specific soil thermometer (HANNA Digital thermometer, HI 98501). Three random measurements were performed inside and outside the forest canopy. The measured values were subsequently averaged and used for the calculation of the ∆T, that is, the difference between the average temperatures of the topsoil outside the forest canopy and of the topsoil under the forest canopy.

### 4.5. Data Analysis

The data were processed using the “vegan” package [64] of the R software [65]. To highlight the pattern of species composition of the two forest coenoses types we merged the vascular plant taxa occurring in more than one layer. In the resulting data set, each species was present once. Before calculations, the ecological variables matrix was undergone at a normalization process using the “decostand” function on the vegan package. To determine if the plot types had different community compositions, we conducted a nonmetric multidimensional scaling (NMDS) analysis with Bray-Curtis distance measure. Indicator species analysis (ISA) [66] was performed to identify the indicator species for each group. These species were identified for each group using the indicator value (IndVal) method, which combines the specificity of a species (uniqueness to a particular sampling unit) and its fidelity (frequency within that sampling unit). For each species, the IndVal ranges from 0 (no indication) to 1 (maximum indication). The statistical significance of the IndVal was tested using a Monte Carlo test, based on 999 randomizations. To highlight differences between vegetation layers of the two forests coenoses we calculated species Richness and Shannon diversity index for the tree, shrub and herb layers of each plot. Then we performed the analysis of variance and tested the significance among the averages of the two groups. The ecological determinants of the species composition and richness of the two forest types were investigated by redundancy analysis (RDA). The RDA allowed the comparison of the forests and the identification of the gradient trajectories for the environmental variables. Only variables that showed a significant difference among *A. altissima* and native forests were used for the model. The redundancy analysis was performed on the Hellinger transformed vegetation matrix. The Monte Carlo permutation test (based on 999 iterations) was performed in order to assess both the significance of the environmental variables and the ordination axes. Box plot diagrams were used to illustrate data distribution of ecological variable and species characteristics. To analyze the variance of the groups and tests for significance we used ANOVA (“aov” function of “stats” package). The Shapiro test was used to test the normality of the analyzed data and the Bartlett test for homoscedasticity.

## 5. Conclusions

The alien forest communities were located between 10 and 500 m above the sea level, with an average age > 20 years, an area ranging from 300 m^2^ up to 3000 m^2^, and an average canopy height of 12 m. The comparison of the floristic composition, diversity, and environmental factors between *A. altissima* coenoses and native forest coenoses, highlighted that the main differences were present at the level of the herbaceous layer. Interestingly, *A. altissima* forests are characterized by higher species richness in the herbaceous layer, constituted by annual and perennial herbaceous species (therophytes and hemicryptophytes), with ruderal strategy and linked to disturbed conditions. Moreover, there is a total absence of a pool of nemoral species found in the native forests that are an indicator of habitat quality. The difference detected in species richness could be linked to changes in environmental variables, in fact, *A. altissima* coenoses showed lower total nitrogen and carbon and C/N ratio values due to an accelerated nutrient cycling process. Our findings highlight that immediate management plans are needed to protect the native plant communities. Further analysis is needed to better understand the below-ground processes and the effect of the litter layer and allelopathy related to this invasive species on the resident plant community.

## Figures and Tables

**Table 1:** Comparison of the vegetation layers. Mean values of the species richness and Shannon diversity index relative to the *A. altissima* and native forests for the tree, shrub, and herbaceous layers. Results from the ANOVA test between the two groups and the significance level is given. Significant values are in bold.

		*Ailanthus* Forests	Native Forests	*Ailanthus* Vs. Native	
		Mean	Mean	Mean sq	*p*-Value
Species richness	tree layer	2.42	2.95	2.63	0.18
Species richness	shrub layer	7.16	8.84	13.92	0.25
Species richness	herb-layer	8.16	7.74	50.95	0.05
Shannon index	tree layer	0.68	0.89	0.41	0.18
Shannon index	shrub layer	1.81	2.00	0.34	0.19
Shannon index	herb-layer	2.13	1.91	0.45	0.16

**Table 2:** Redundancy analysis. Ordination parameters of redundancy analysis (RDA). Significance of the environmental factors. *p* level: *** *p* < 0.001; ** *p* < 0.01; * *p* ≤ 0.05.

Ecological Variables	df	Variance	F	*p*	
N_tot	1	0.02694	1.69	0.012	*
C_tot	1	0.03234	2.03	0.001	***
C/N	1	0.01138	0.72	0.896	
CC	1	0.01431	0.90	0.596	
CH	1	0.03037	1.91	0.005	**
ΔPAR_soil	1	0.01163	0.73	0.891	
ΔT_soil	1	0.02625	1.65	0.020	*
Residual	30	0.47722			

**Table 3:** Measured variables. Description of the topographic characteristics and ecological variables considered, with the indication of the unit and the respective symbol for each variable.

Topographic and Ecological Variables	Unit	Symbol
Altitude	m a.s.l.	Alt
Slope	°	Sl
Northness	°	N
Canopy Cover	%	CC
Canopy Height	m	CH
Photosynthetic active radiation at 1.30 m from the soil	(µmol m^−2^ s^−1^)	PAR_chest
Photosynthetic active radiation at the soil level	(µmol m^−2^ s^−1^)	PAR_soil
Photosynthetic active radiation in full light condition	(µmol m^−2^ s^−1^)	PAR_out
Temperature of the topsoil under forest canopy	°C	T_in
Temperature of the topsoil outside forest canopy	°C	T_out
Temperature of the air	°C	T_air
Difference PAR_out-PAR_chest		Δ PAR_chest
Difference PAR_out-PAR_soil		Δ PAR_soil
Difference T_out-T_in		Δ T soil
Total nitrogen	g/kg	N_tot
Total extractable carbon	g/kg	C_tot
carbon-nitrogen ratio	g/kg	C/N
pH in H_2_O	pH unit	pH

## Supplementary Materials

The following are available online at https://www.mdpi.com/2223-7747/9/10/1404/s1, Table S1: A list of species recorded in the study area, Table S2: Minimum, average and maximum values of the topographic characteristics and ecological variables surveyed in the *A. altissima* forests and native forests, Table S3: Geographical coordinates of the vegetation plots. Coordinates system WGS84-UTM33.
