# Supplementary material for: Ailanthus altissima Forests Determine a Shift in Herbaceous Layer Richness: A Paired Comparison with Hardwood Native Forests in Sub-Mediterranean Europe
Source: Plants (Basel). 2020 Oct 21;9(10):1404. doi: 10.3390/plants9101404 (PMC7589998; doi:10.3390/plants9101404)
Supplement: Supplementary file 1 [file plants-09-01404-s001.pdf]

plants MDPI

Supplementary Materials to the paper Montecchiari, S. Tesei, G. Allegrezza, M. *Ailanthus altissima* forests determine a shift in herbaceous layer richness: a paired comparison with native forests in sub Mediterranean Europe.

Table S1. A list of species recorded in the study area. Indication of percentage frequency values for each species present respectively in the *A. altissima* forest plots (%AIL) and native forest plots (%NAT), life form, Grime life strategy, and indication of nemoral status (nem_herb). At the end of the table are indicated percentage frequencies of seedlings and saplings recorded in *A. altissima* forests and Native forests. Nomenclature follows Bartolucci et al.,2018.

Species	%AIL	%NAT	Life Form	Grime Strategy	Nemoral Status
Acer campestre L.	52,6	63,2	P scap	C	
Acer pseudoplatanus L.	5,3		P scap	C	
Aegonychon purpurocaeruleum (L.) Holub		10,5	H scap	CS	Nem_herb
Ailanthus altissima (Mill.) Swingle	100,0	15,8	P scap	C	
Alliaria petiolata (M.Bieb.) Cavara & Grande	15,8	15,8	H scap	CR	
Allium sp.	5,3				
Anisantha sterilis (L.) Nevski	31,6	5,3	T scap	CR	
Artemisia campestris L.	5,3		Ch suffr	CS	
Arum italicum Mill. subsp. *italicum*	5,3	10,5	G rhiz	SR	
Arundo donax L.	5,3	10,5	G rhiz	SR	
Asparagus acutifolius L.	26,3	31,6	G rhiz	CS	
Avena barbata Pott ex Link	5,3		T scap	SR	
Ballota nigra L. subsp. *nigra*		5,3	H scap	C	
Bellevalia romana (L.) Sweet	5,3		G bulb	CS	
Brachypodium rupestre (Host) Roem. & Schult.	10,5	26,3	H caesp	CS	
Brachypodium sylvaticum (Huds.) P.Beauv.	15,8	21,1	H caesp	CS	
Bunium bulbocastanum L.	5,3		G bulb	CSR	
Campanula trachelium L. subsp. *trachelium*		5,3	H scap	CS	Nem_herb
Carex flacca Schreb. subsp. *flacca*		10,5	G rhiz	CSR	
Carex pendula Huds.	5,3	5,3	H caesp	CS	Nem_herb
Celtis australis L. subsp. *australis*		5,3	P scap	CSR	
Cercis siliquastrum L. subsp. *siliquastrum*	10,5	5,3	P scap	CSR	
Chaerophyllum temulum L.	31,6		T scap	CR	
Clematis vitalba L.	78,9	52,6	P lian	C	
Clinopodium nepeta (L.) Kuntze	15,8	5,3	H scap	C	
Clinopodium vulgare L. subsp. *vulgare*	5,3		H scap	CS	
Convolvulus arvensis L.	5,3		G rhiz	CR	
Cornus sanguinea L.	47,4	36,8	P caesp	C	
Corylus avellana L.	5,3		P caesp	C	
Crataegus laevigata (Poir.) DC.		10,5	P caesp	C	
Crataegus monogyna Jacq.	57,9	68,4	P caesp	C	
Cruciata glabra (L.) C.Bauhin ex Opiz	10,5	5,3	H scap	CSR	
Cruciata laevipes Opiz	5,3		H scap	CSR	
Cyclamen hederifolium Aiton		10,5	G bulb	CSR	Nem_herb
Cyclamen repandum Sm. subsp. *repandum*		5,3	G bulb	CSR	Nem_herb
Cytisophyllum sessilifolium (L.) O.Lang		5,3	P caesp	CSR	
Dactylis glomerata L. subsp. *glomerata*	10,5	5,3	H caesp	C	
Daucus carota L. subsp. *carota*	5,3		H bien	CR	
Dioscorea communis (L.) Caddick & Wilkin	5,3	5,3	G rad	C	
Elymus repens (L.) Gould subsp. *repens*	5,3	10,5	G rhiz	S	
Emerus major Mill. subsp. *emeroides* (Boiss. & Spruner) Soldano & F.Conti		10,5	Np	CS	

Euonymus europaeus L.	47,4	47,4	P caesp	C	
Ficus carica L.	5,3	10,5	P scap	CS	
Fraxinus ornus L. subsp. *ornus*	26,3	36,8	P scap	C	
Galium album Mill. subsp. *album*	15,8	10,5	H scap	C	
Galium aparine L.	21,1	5,3	T scap	CR	
Galium mollugo L.	5,3		H scap	C	Nem_herb
Geranium dissectum L.	5,3		T scap	CR	
Geranium robertianum L.		5,3	T scap	CSR	Nem_herb
Geranium sp. pl.	5,3				
Geum urbanum L.	5,3		H scap	CSR	
Hedera helix L. subsp. *helix*	84,2	89,5	P lian	CS	
Helleborus foetidus L. subsp. *foetidus*	5,3		Ch suffr	CS	
Helleborus viridis L. subsp. *bocconei* (Ten.) Peruzzi		5,3	G rhiz	CS	Nem_herb
Inula conyzae (Griess.) DC.	5,3		H bien	CS	
Iris germanica L. s.l.	5,3		G rhiz	C	Nem_herb
Juglans regia L.	5,3	10,5	P scap	C	
Lamium maculatum L.	5,3		H scap	CSR	
Lamium sp.	15,8	15,8			
Lapsana communis L. subsp. *communis*	5,3		T scap	CR	
Laurus nobilis L.	52,6	47,4	P caesp	C	
Ligustrum lucidum W.T.Aiton	5,3	10,5	Np	CS	
Ligustrum vulgare L.	15,8	36,8	Np	C	
Lonicera caprifolium L.	5,3	5,3	P lian	C	
Lonicera etrusca Santi		21,1	P lian	C	
Lunaria annua L.	5,3	5,3	H scap	CR	
Malus sp.	5,3		P csesp		
Melica uniflora Retz.		5,3	H caesp	C	Nem_herb
Melissa officinalis L. s.l.	47,4	15,8	H scap	C	
Mentha spicata L.	5,3		H scap	C	
Narcissus sp.	5,3				
Olea europaea L.	21,1	5,3	P caesp	CS	
Origanum vulgare L. subsp. *vulgare*	5,3		H scap	CSR	
Orobanche minor Sm.		10,5	T scap	SC	Nem_herb
Ostrya carpinifolia Scop.		31,6	P caesp	C	
Osyris alba L.	10,5	15,8	Np	SR	
Paliurus spina-christi Mill.	15,8	10,5	P caesp	CS	
Parietaria judaica L.	5,3		H scap	CSR	
Petrosedum rupestre (L.) P.V.Heath		5,3	Ch succ	SR	
Phillyrea latifolia L.		10,5	P caesp	CS	
Picris hieracioides L. subsp. *hieracioides*	10,5		H scap	CSR	
Pistacia terebinthus L. subsp. *terebinthus*		5,3	P caesp	CS	
Poa trivialis L.	15,8		H caesp	CSR	
Populus alba L.	5,3		P scap	C	
Populus canescens (Aiton) Sm.		5,3	P scap	C	
Populus nigra L. subsp. *nigra*	10,5	5,3	P scap	C	
Primula vulgaris Huds. subsp. *vulgaris*		10,5	H ros	CSR	Nem_herb
Prunus avium (L.) L.	21,1	36,8	P scap	C	
Prunus domestica L.	10,5	5,3	P caesp	C	
Prunus spinosa L. subsp. *spinosa*	42,1	15,8	P caesp	C	
Pseudoturritis turrita (L.) Al-Shehbaz	5,3	15,8	H bien	CR	
Pyrus communis L. subsp. *pyraster* (L.) Ehrh.	5,3	5,3	P scap	C	
Quercus cerris L.		10,5	P scap	C	
Quercus ilex L. subsp. *ilex*	5,3	10,5	P scap	C	
Quercus pubescens Willd. subsp. *pubescens*	36,8	63,2	P caesp	C	
Rhus coriaria L.		5,3	P caesp	C	

Robinia pseudoacacia L.	15,8	36,8	P caesp	C	
Rosa canina L.	10,5	15,8	Np	CRS	
Rosa sempervirens L.	5,3		Np	C	
Rosa sp.		5,3	Np		
Rubia peregrina L.	26,3	36,8	P lian	CS	
Rubus caesius L.	5,3	5,3	Np	C	
Rubus ulmifolius Schott	84,2	68,4	Np	CS	
Rumex sanguineus L.	5,3		H scap	CS	
Ruscus aculeatus L.	5,3	10,5	Ch frut	S	Nem_herb
Sambucus nigra L.	42,1	15,8	P caesp	C	
Silene italica (L.) Pers.	10,5	5,3	H ros	C	
Silene latifolia Poir.	5,3		H bien	C	
Sonchus asper (L.) Hill subsp. *asper*	5,3		T scap	CR	
Sorbus domestica L.		5,3	P scap	C	
Spartium junceum L.	5,3		P caesp	CR	
Stachys sylvatica L.	5,3	5,3	H scap	CS	
Stellaria media (L.) Vill. subsp. *media*	10,5		T rept	CR	
Symphyotrichum squamatum (Spreng.) G.L.Nesom	5,3		T scap	CS	
Teucrium chamaedrys L. subsp. *chamaedrys*		5,3	Ch suffr	CSR	
Torilis arvensis (Huds.) Link subsp. *arvensis*	21,1		T scap	CR	
Ulmus minor Mill. subsp. *minor*	36,8	52,6	P caesp	C	
Umbilicus horizontalis (Guss.) DC.	5,3		G bulb	SR	
Urtica dioica L. subsp. *dioica*	26,3	5,3	H scap	C	
Verbascum thapsus L. subsp. *thapsus*	5,3		H bien	C	
Viburnum tinus L. subsp. *tinus*	5,3	10,5	P caesp	CSR	
Viola alba Besser subsp. *dehnhardtii* (Ten.) W.Becker	15,8	21,1	H ros	CSR	Nem_herb
Vitis vinifera L.		10,5	P lian	C	
**Seedlengs and Saplings**					
Ailanthus altissima (Mill.) Swingle pl	73,7	5,3			
Laurus nobilis L. pl	31,6	31,6			
Quercus pubescens Willd. pl	26,3	47,4			
Acer campestre L. pl	21,1	36,8			
Ulmus minor Miller pl	5,3	21,1			
Fraxinus ornus L. pl	5,3	15,8			
Ligustrum vulgare L. pl	5,3	10,5			
Pyrus communis L. pl	5,3	5,3			
Quercus ilex L. pl	5,3	5,3			
Sambucus nigra L. pl	15,8	5,3			
Crataegus monogyna Jacq. pl	21,1				
Euonymus europaeus L. pl	15,8				
Ligustrum lucidum W.T.Aiton pl	5,3				
Populus alba L. pl	5,3				
Cercis siliquastrum L. pl		5,3			
Cornus sanguinea L. pl		5,3			
Ostrya carpinifolia Scop. pl		5,3			
Populus canescens (Aiton) Sm. pl		5,3			
Prunus avium (L.) L. pl		5,3			
Quercus cerris L. pl		5,3			
Rosa canina L. pl.		5,3			
Sorbus domestica L. pl.		5,3			
Viburnum tinus L. pl		5,3			

Table S2. Minimum, average and maximum values of the topographic characteristics and ecological variables surveyed in the *A. altissima* forests and native forests.

	**Ailanthus Forests**			**Native Forests**		
	Min	Average	Max	Min	Average	Max
Alt (m slm)	9	194	462	18,89	192	463
Slope (°)	1	10	23	0	10	33
Northness	-1	0	1	-1	0	1
Sup (m2)	215	833	3045	-	>2000	-
Canopy_cover (%)	74	88	94	61	88	97
Canopy_height (m)	7,4	13,4	21,6	6	14,5	24,8
PAR_chest (μmol m-2s-1)	3,2	25,8	97,1	7,3	28,2	64,4
PAR_soil (μmol m-2s-1)	2,2	21,4	125,5	6,1	19,8	49,8
PAR_out (μmol m-2s-1)	83,1	1349,2	1906,8	83,1	1213,1	1863,3
T_in (°C)	15,7	18,9	22,3	15,8	18,4	20,2
T_out (°C)	18,1	21,7	25,6	17,4	21,2	29,2
T_air (°C)	19,2	25,8	29,5	19	25,4	32,3
Δ PAR_chest	61,2	1292,7	1875,3	71,1	1184,9	1854,5
Δ PAR_soil	66,2	1297,1	1874,1	75,5	1193,3	1856
Δ T	-0,5	2,7	7,9	-0,7	2,8	9,5
N_tot (g/Kg)	1,5	2,8	4,8	1,6	3,7	6
C_tot (g/Kg)	5,7	20,8	36,9	11,1	32,2	56,9
C/N (g/Kg)	3,8	7,3	9	6,9	8,5	10,2
pH	7,4	7,8	8,1	6,2	7,6	7,9

Table S3. Geographical coordinates of the vegetation plots. Coordinates system WGS84-UTM33.

**ID**	**East**	**North**
5	385604	4814288
7	376931	4799761
8	375047	4796378
9	346354	4816786
11	339474	4796335
12	337269	4859623
14	375354	4813757
15	372499	4805753
16	337494	4796349
17	334511	4797268
18	333757	4797518
19	356709	4779328
20	349941	4777643
21	338904	4811611
22	341462	4824278
23	337132	4853921
25	337141	4848681
28	329718	4844106
29	321483	4839316
5 Nat	385786	4813831
7 Nat	376900	4799729
8 Nat	375913	4797043
9 Nat	346288	4816722
11 Nat	334511	4797268
12 Nat	337061	4859794
14 Nat	375487	4813711
15 Nat	372552	485785
16 Nat	337596	4796311
17 Nat	334874	4797533
18 Nat	333769	4797522
19 Nat	356776	4779481

20 Nat	350414	4777647
21 Nat	338860	4811557
22 Nat	341453	4824259
23 Nat	337092	4853935
25 Nat	337222	4848771
28 Nat	329777	4844203
29 Nat	321615	4839182
